# Staphylococcal poisoning during a village festival, Medina, Misamis Oriental, Philippines in 2014

**DOI:** 10.5365/wpsar.2017.8.2.005

**Published:** 2019-05-21

**Authors:** John Bobbie Roca, Ruth Alma Ramos, Herdie Hizon, Vikki Carr de los Reyes, Ma Nemia L Sucaldito, Enrique Tayag

**Affiliations:** a Department of Health, Manila, Philippines.

## Abstract

**Introduction:**

On 18 August 2014, cases of food poisoning in San Vicente Village were reported to the Event-Based Surveillance & Response Unit of the Philippine Department of Health. An investigation was conducted to identify the implicated source, describe the outbreak and evaluate the risk factors.

**Methods:**

A case-control study was conducted. A suspected case was a previously well individual of Medina who attended the village festival and developed abdominal pain and vomiting with or without nausea, diarrhoea and fever from 18 to 19 August. A confirmed case was a suspected case with a rectal swab positive for bacterial culture. Rectal swabs, water and food samples were sent to the national reference laboratories. Food source and consumption interviews and environmental inspections were conducted.

**Results:**

Sixty-four cases and 123 unmatched controls were identified. The median incubation period was 1 hour 15 minutes. Five cases (8%) were positive for *Staphylococcus aureus,* one (2%) for *Aeromonas hydrophilia* and one (2%) for *Shigella boydii.* One (14%) water sample was positive for *Aeromonas spp.* Of the collected food samples, beef steak was positive for *Staphylococcus aureus*. Risk factors were consumption of Filipino-style beef stew (odds ratio [OR]: 6.62; 95% confidence interval [CI]: 2.90–15.12) and stir-fried noodles (OR: 3.15; 95% CI: 1.52–6.50). Prolonged serving time and improper food storage were noted.

**Discussion:**

In this foodborne outbreak, *Staphylococcus aureus* was the likely causative agent. Meals were contaminated due to improper food handling practices. We recommend that a policy be created to mandate that village-appointed food handlers undergo food safety training.

## Introduction

*Staphylococcus aureus* is a Gram-positive bacterium that is predominantly associated with food poisoning (*1*) and causes one of the most common foodborne illnesses worldwide. (*2*) About 25% of healthy people are carriers of *Staphylococcus aureus*. The bacterium is associated with skin, eye, nose or throat infections. (*3*) The most common way for food to be contaminated by the bacteria is through contact with infected food handlers. Other food contamination sources are the equipment or surfaces on which food is prepared (*3*) and infected house flies. (*4*) When food is contaminated, bacteria quickly multiply at room temperature and produce a fast acting enterotoxin (*5*) that can cause nausea, abdominal pain, vomiting and diarrhoea. (*1*)

On 18 August 2014, the Event-Based Surveillance & Response Unit of the Philippine Department of Health received a report of food poisoning among villagers of San Vicente Village, a rural village in Medina, Misamis Oriental on the island of Mindanao. The village is subdivided into seven areas and has a total population of 978. (*6*) Every 18 August, the village celebrates its founding with a festival with free meals for all community members.

A team from the Philippines Field Epidemiology Training Program was deployed to conduct an epidemiologic investigation to identify the implicated source, describe the outbreak and evaluate the risk factors.

## METHODOLOGY

### Epidemiologic investigation

A descriptive study was conducted by reviewing medical records of outpatients and inpatients at the local hospitals. A suspected case was defined as a previously well individual of Medina, Misamis Occidental who attended the village festival and developed abdominal pain and vomiting with or without nausea, diarrhoea and fever from 18 to 19 August 2014. A confirmed case was a suspected case with a positive rectal swab in bacterial culture.

An unmatched case-control study was conducted. Controls were individuals of Medina who attended the village festival and did not develop any symptoms and were negative on bacterial stool cultures. Subjective sampling from suspected and confirmed patient lists was used to identify cases for the study. Controls were identified from the same household and/or nearby households of the cases. Cases and controls were interviewed using a standard questionnaire that included demographics, symptoms (except for controls), history of food consumption within the past 24 hours, source of drinking-water, hygiene practices and other environmental factors.

Statistical analysis, including calculation of odds ratios (OR) and 95% confidence intervals (CI), was done using EpiInfo version 3.5.4 software. Significant bivariate analysis results were then tested by multivariable analysis.

### Laboratory examinations

Rectal swabs were collected for culture and sensitivity testing from cases and controls including food handlers. Water samples from the water reservoir and communal faucets were collected for bacteriologic analysis. Both were sent to the national reference laboratories. Food samples were sent to the Food and Drug Administration Satellite Laboratory for Mindanao for bacteriologic analysis.

### Environmental investigation

We visited the food handling and preparation area and water sources. We interviewed food handlers on the food production chain, food consumption history and presence of signs and symptoms.

### Ethical approval

Ethics clearance was not required according to local regulations as this investigation was part of an emergency response to an outbreak. However, a signed consent was obtained before interviews and specimen collection.

## Results

### Case-control study

All 64 cases (57 suspected and seven confirmed) and 123 controls were included in the study. All cases and 121 controls ate food served at the festival. Six out nine food handlers were included in the case-control study. One food handler did not meet the definition of case or control, and two others could not be located for the study. All of the interviewed food handlers fit the control definition. All of the individuals approached agreed to be involved in the study.

**Fig. 1:**
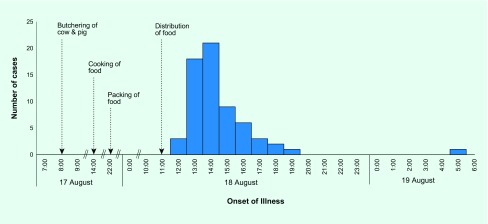
Distribution of foodborne illness cases by onset of illness (*n* = 64), San Vicente Village, Medina, Misamis Oriental, Philippines, 18–19 August 2014

The first case manifested signs and symptoms in less than 15 minutes after ingestion of food. The number of subsequent cases peaked by 14:00. The median incubation period was 1 hour 15 minutes (range: 10 minutes to 16.98 hours). No deaths were reported (**Fig. 1**). All cases had abdominal pain and vomiting. Other symptoms reported were nausea (88%), diarrhoea (52%) and fever (16%). There were 40 (63%) female cases; ages ranged from 1 to 75 years (median: 22 years, interquartile range: 7 to 38 years). The most affected age group was 21–35 years (25%).

Bivariate analysis revealed that consumption of Filipino-style beef stew (OR: 8.16, 95% CI: 3.77–17.66) and stir-fried noodles (OR: 2.38; 95% CI: 1.28–4.40) were risk factors for food poisoning (**Table 1**). After adjusting for demographics and exposure variables, consumption of Filipino-style beef stew (OR: 6.62; 95% CI: 2.90–15.12) and stir-fried noodles (OR: 3.15; 95% CI: 1.52–6.50) remained statistically significant risk factors for food poisoning. On the contrary, consumption of pork humba (OR: 0.42; 95% CI: 0.20–0.89) and Filipino-style pork stew (OR: 0.22; 95% CI: 0.06–0.83) were inversely associated with being a case.

**Table 1:** Factors associated with staphylococcal foodborne illness cases, San Vicente Village, Medina, Misamis Oriental, Philippines, 18–19 August 2014

Factors	Case *n*(%)	Control *n*(%)	Crude OR (95% CI)	Adjusted OR (95% CI)
**Sex**				
Male	24 (37)	63 (51)	0.57 (0.31–1.06)	-
Female	40 (63)	60 (49)		
Type of food consumed*				
Filipino-style beef stew	30 (47)	12 (10)	8.16 (3.77–17.66)	6.62 (2.90–15.12)
Stir-fried noodles	37 (58)	45 (37)	2.38 (1.28–4.40)	3.15 (1.52–6.50)
Beef innards stew	2 (3)	3 (2)	1.29 (0.11–11.60)	-
Beef curry	7 (11)	11 (9)	1.25 (0.46–3.40)	-
Pork curry	0 (0)	0 (0)	-	-
Rice	47 (73)	91 (74)	0.97 (0.49–1.93)	-
Beef steak	33 (51)	80 (65)	0.57 (0.31–1.06)	-
Pork humba	19 (30)	62 (50)	0.42 (0.22–0.79)	0.42 (0.20–0.89)
Filipino-style pork stew	3 (5)	27 (22)	0.17 (0.03–0.61)	0.22 (0.06–0.83)
**Other factors**				
Washed hands before eating	63 (98)	118 (96)	1.06 (0.09–11.90)	-
Used both spoon and fork to eat	60 (94)	116 (94)	0.52 (0.09–2.89)	-
Boiled drinking-water	25 (39)	70 (57)	0.49 (0.26–0.90)	-
Washed hands after toilet use	62 (97)	119 (97)	0.26 (0.02–2.91)	-
Consumed packed lunch	64 (100)	121 (98)	-	-
Drank from communal faucet	64 (100)	123 (100)	-	-

OR, odds ratio; CI, confidence interval.

* May have had more than one response.

### Laboratory examinations

A rectal swab was collected from each of the 64 cases and 123 controls. Of the samples from cases, five (8%) were positive for *Staphylococcus aureus,* one for (2%) for *Aeromonas hydrophilia* and one (2%) for *Shigella boydii.* However, 45 (70%) of the cases were given antibiotics before specimen collection. All controls showed no growth in the bacterial culture test. One out of the seven (14%) rectal swab cultures from food handlers was positive for *Aeromonas sobria.*

One out of the eight (13%) water samples collected was found to be positive for *Aeromonas species.*

Beef steak and rice were the only leftover food samples collected. Bacterial culture revealed that the beef steak was positive for *Staphylococcus aureus;* the culture from the rice yielded no bacterial growth.

### Environmental investigation

Seven out of the nine village-appointed food handlers were interviewed. All were asymptomatic. Food source investigation revealed that a cow and a pig were bought from a local farm, while the vegetables and commercially prepared seasonings came from a nearby market. Animals were slaughtered in an open space at the town hall by 08:00 on 17 August 2014 (the day before consumption). Meat and entrails were butchered to desired cuts. Cooking of dishes started by 14:00 with beef dishes prepared first followed by the pork dishes. The cooking process ended by 22:30. Water from communal faucets was used to wash raw ingredients and for cooking. Cooked dishes were cooled in a separate room and covered with banana leaves.

**Fig. 2:**
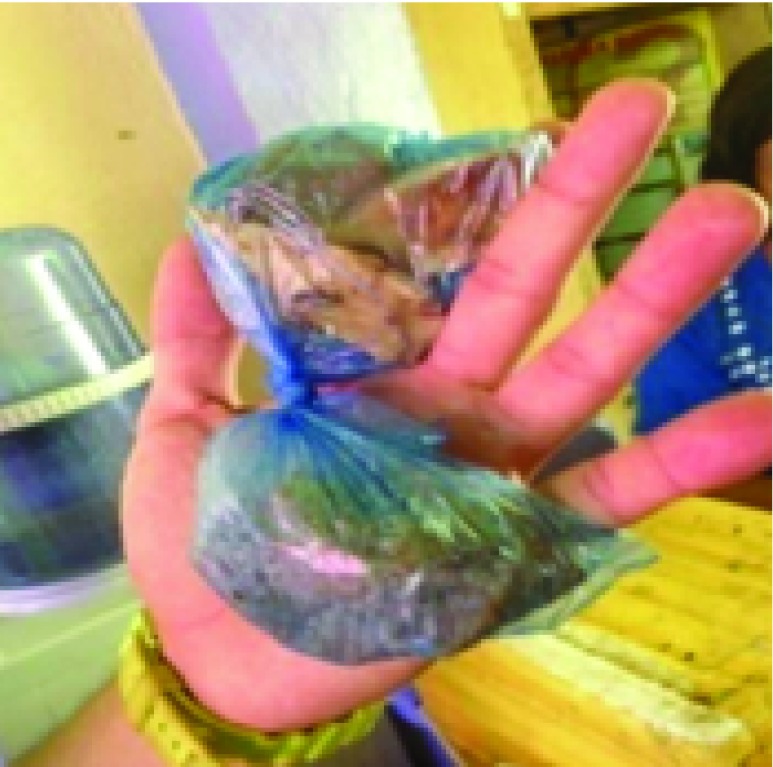
Packed meal

Meals were packed between 22:45 on 17 August 2014 and 06:00 the following day. Two varieties of dishes were packed in a “chorizo-like” manner where one plastic bag was used (**Fig. 2**). Packed meals were stored in either a plastic tray, carton box or empty rice sack at room temperature.

By 11:30, packed meals were distributed among villagers.

No food handlers wore aprons or hair nets during food preparation. They did not have formal food safety training, and proper hand hygiene was not observed. Flies were also claimed to be present during food preparation.

No chlorine residue was noted inside the water reservoir, and breakage in water distribution pipelines was seen.

## Discussion

The epidemiological evidence suggests that the most likely source of this foodborne outbreak was the consumption of contaminated packed meals served during the village festival. The short incubation period (median 1 hour 15 minutes) and the symptoms manifested by cases suggest a *Staphylococcus aureus* enterotoxin poisoning. *Staphylococcus aureus* was seen in the human specimens and food samples (beef steak); both consumption of Filipino-style beef stew and stir-fried noodles were statistically most likely to be associated with the illness.

The issue of food safety practices by the food handlers played a part in this outbreak. The observed improper food handling practices such as poor hand washing technique, prolonged serving time (*2*, *7*) and improper temperature for food storage (*1*, *2*, *5*, *7*) have been linked to staphylococcal foodborne outbreaks.

The isolation of *Aeromonas hydrophilia* and *Shigella boydii* in one of the cases could have been incidental to this outbreak. The typical incubation period of 12 to 72 hours (*1*) after ingestion of food contaminated by both bacteria does not coincide with the incubation period of the cases.

The Sanitation Code of the Philippines requires all food caterers, regardless of type and enterprise size, to secure sanitary permits and health certificates for all their employees before operation. (*8*, *9*) This policy only covers licensed food establishments. However, most foodborne outbreaks in the Philippines occur in home settings and at events where the food handlers are not trained on food safety. (*10*)

This study has some limitations. First, we were not able to locate and test all the food handlers. Second, most the cases were already treated with antibiotics before stool collection. This may have contributed to low positivity rates in clinical specimens. Third, dose–response was not investigated. Fourth, there is the possibility of recall bias on the specific food exposure due to the retrospective nature of data finding. In spite of these limitations, we were able to identify the source of this outbreak from both the clinical and epidemiological results.

As a response to the outbreak, we recommended the reinforcement of the Sanitation Code of the Philippines by municipal governments through the release of an ordinance mandating that village-appointed food handlers secure updated health certificates and attend formal food safety training before engaging in mass feeding activities to prevent further outbreaks.
